# GAAIS-J: Translation and Validation of the Japanese Version of the General Attitudes Toward Artificial Intelligence Scale

**DOI:** 10.3390/bs15121668

**Published:** 2025-12-03

**Authors:** Yasumasa Yamaguchi, Chiaki Hashimoto, Nagayuki Saito

**Affiliations:** Department of Sports Intelligence & Mass Media, Sendai University, 2-2-18, Funaoka Minami, Shibata-machi, Shibata-Gun 989-1693, Miyagi-ken, Japan

**Keywords:** artificial intelligence attitudes, scale adaptation and validation, psychometrics

## Abstract

The rapid integration of artificial intelligence (AI) into daily life highlights the need for culturally adapted tools to assess public attitudes. This study translated, culturally adapted, and validated the Japanese version of the General Attitudes toward Artificial Intelligence Scale (GAAIS-J) to enable cross-cultural research. The original GAAIS was translated, back-translated, and reviewed by experts for cultural and linguistic equivalence. A web-based survey of 3689 Japanese was conducted. Confirmatory factor analysis supported a two-factor model—positive and negative attitudes—after removing one low-loading item and allowing select item covariances, yielding acceptable fit indices. Reliability was high (ω = 0.94, α = 0.92), and average variance extracted values indicated satisfactory convergent validity. Construct validity was demonstrated through correlations with AI trust/distrust, generative AI usage frequency, socioeconomic status, gender, and Big Five traits. Positive attitudes were linked to higher trust, income, and openness; negative attitudes correlated with older age, lower education, and lower agreeableness. The GAAIS-J is a reliable, valid instrument for assessing AI attitudes in Japan and supporting international comparisons.

## 1. Introduction

Artificial Intelligence (AI) is being increasingly integrated into various domains such as healthcare, finance, education, and entertainment, reshaping societal structures and everyday life (Dixon, 2024). As the influence of AI expands, understanding public attitudes toward it becomes critical for its ethical development, responsible deployment, and social acceptance. One established tool for measuring attitudes toward AI is the General Attitudes toward Artificial Intelligence Scale (GAAIS), developed by Schepman and Rodway (2023). The GAAIS captures both positive and negative perceptions of AI and has demonstrated strong psychometric properties in prior research.

The GAAIS employs a two-factor structure—positive and negative attitudes—enabling comprehensive assessment of cognitive and emotional evaluations of AI (Schepman & Rodway, 2023). It has been associated with various psychosocial variables such as personality traits, political orientation, and risk perception, and shows external validity through its relationships with AI trust and usage experiences (Fast & Horvitz, 2017; Zhang & Dafoe, 2019).

Given increasing interest in cross-cultural AI perception, adapting such instruments requires more than linguistic translation. Cultural context significantly shapes AI-related attitudes, and establishing construct equivalence through cross-cultural validation is essential (F. J. R. Van de Vijver & Leung, 1997; Tussyadiah, 2020). Thus, translating and validating international psychological instruments in the Japanese context is a critical step toward advancing research on attitudes toward AI.

Despite the growing relevance of AI in Japan, no standardized Japanese version of the GAAIS currently exists. Previous research has suggested that Japanese attitudes toward AI and robotics differ from those in Western societies, often showing greater affinity (Nomura et al., 2006; Nomura et al., 2008). Developing a culturally adapted, psychometrically sound version of the GAAIS would thus facilitate both domestic and international comparative studies.

The present study aimed to translate and culturally validate the GAAIS for use in Japan, examining structural equivalence and external validity while maintaining fidelity to the original instrument. Through a rigorous translation and back-translation process and confirmatory factor analysis, this study assesses the structural validity of the GAAIS-J. Further, associations with theoretically related constructs—including AI trust/distrust, generative AI usage frequency, and Big Five personality traits—are examined to evaluate external validity. The validated GAAIS-J is expected to serve as a standard tool for assessing AI attitudes in Japan and contribute to cross-cultural research in human–AI interaction.

## 2. Literature Review

Attitudes toward AI are grounded in established theories from technology acceptance and trust research. The Technology Acceptance Model (TAM) emphasizes perceived usefulness and ease of use as predictors of technology attitudes (Davis, 1989). Studies applying TAM to AI show that usefulness strongly predicts AI adoption, while ease of use varies in effect (Schepman & Rodway, 2023). The Unified Theory of Acceptance and Use of Technology (UTAUT) expands TAM by adding performance expectancy, effort expectancy, social influence, and facilitating conditions, moderated by user traits (Venkatesh et al., 2003). Both models explain how functional appraisals and contextual factors shape AI attitudes. Trust theory further enriches this understanding. Trust in AI—defined as willingness to rely on AI under uncertainty—depends on perceived competence, integrity, and benevolence (Glikson & Woolley, 2020). High trust correlates with acceptance, while distrust limits uptake. Together, these models frame attitudes as shaped by rational appraisals and emotional evaluations.

Culture moderates AI perceptions. Japanese society is often described as more accepting of AI, influenced by animism and media (Nomura et al., 2006). While early studies showed differences in robot attitudes (Bartneck et al., 2010), later work suggested more cross-cultural similarity than assumed (MacDorman et al., 2009). Nonetheless, cultural framing matters. Japanese individuals tend to ascribe more emotional and autonomous qualities to AI (Nomura et al., 2006), while Westerners focus on practical utility. Recent studies show that similar psychological factors predict AI attitudes in both Japan and the West, including job loss anxiety and AI familiarity (Persson et al., 2021). Cultural narratives still color perception, but universal drivers such as trust, exposure, and perceived threat are influential across contexts.

Several instruments have been developed to assess attitudes toward AI. The General Attitudes toward Artificial Intelligence Scale (GAAIS) captures both positive and negative sentiments (Schepman & Rodway, 2023). The Attitude Toward Artificial Intelligence Scale (ATAI) measures acceptance and fear with five items (Sindermann et al., 2020), and the Threats of AI Scale (TAI) focuses on fear-related aspects (Kieslich et al., 2021). In Japan, the Negative Attitudes toward Robots Scale (NARS) was one of the earliest tools developed to capture public responses to robotics (Nomura et al., 2008), reflecting the country’s longstanding interest in human–robot interaction. More recently, trust-specific instruments have also emerged, including Katase’s (2021) AI Trust Scale, which differentiates between distrust in AI’s fidelity and trust in its social utility. Outside Japan, Jian et al. (2000) proposed a general trust-in-automation scale, and later tools have measured trust’s specific subdimensions (Glikson & Woolley, 2020). Together, these instruments provide a multidimensional view of AI perception across contexts.

Demographic and psychological factors also influence AI attitudes. Age consistently predicts AI perceptions, with younger people showing more optimism (Zhang & Dafoe, 2019). Education and SES correlate with AI familiarity and acceptance (Ipsos, 2022). Gender effects are modest—men often report slightly greater confidence, but attitudinal differences are small when controlling for other factors (Schepman & Rodway, 2023). Personality traits also matter: openness and conscientiousness are linked to positive AI views, while agreeableness predicts trust. Neuroticism shows mixed associations, and extraversion tends to be unrelated (Schepman & Rodway, 2023). Traits such as technophilia and technology anxiety further contribute to individual differences (Sindermann et al., 2020). Cross-national research indicates that these predictors operate similarly across cultures; for instance, job-loss concerns and AI exposure affect attitudes in both Sweden and Japan (Persson et al., 2021). Thus, while certain measurement tools and cultural contexts are specific to Japan, the underlying psychological mechanisms shaping AI attitudes appear broadly shared across societies.

Building upon these findings, the present study aimed to translate and culturally validate the General Attitudes toward Artificial Intelligence Scale (GAAIS) for use in Japan. In doing so, we also examined how demographic and psychological factors—such as age, education, income, and personality traits—relate to individuals’ positive and negative attitudes toward AI. These variables were included not as the main focus but as external criteria to test the construct validity of the adapted scale. By integrating the translation and validation process with an examination of theoretically relevant correlates, this study seeks to provide a comprehensive understanding of AI attitudes in the Japanese context and to contribute to cross-cultural comparisons with the original English version.

## 3. Translation Procedure

Two native Japanese speakers familiar with neuropsychological questionnaires independently translated the original GAAIS items into Japanese. A reconciled version was then created by integrating the two translations. This version was back-translated into English by a bilingual speaker fluent in both Japanese and English. Discrepancies between the original and back-translated versions were reviewed and resolved through consensus, emphasizing conceptual over literal translation to ensure semantic and cultural equivalence.

## 4. Method

A web-based survey was conducted to assess the reliability and validity of the GAAIS-J. Participants were informed that their responses were voluntary, anonymized, and statistically analyzed. Informed consent was obtained before the survey began. Participants were recruited via a survey company that distributed the questionnaire only to eligible monitors. The survey was conducted from 14 to 17 March 2025, yielding 5575 responses. After excluding responses failing attention checks, data from 3689 valid participants were analyzed (2161 males, 1528 females; mean age = 51.65, SD = 11.52). The questionnaire included demographic information, the GAAIS-J, items on generative AI usage frequency, the Big Five personality traits by TIPI-J (Oshio et al., 2012) and Katase’s (2021) trust in AI scale for construct validity testing. The present survey was conducted with the approval of the ethics committee of the first author’s affiliated research institution.

All analyses were conducted using JASP (version 0.19.3), an open-source software for advanced statistical modeling and structural equation analysis.

## 5. Result

### 5.1. Confirmatory Factor Analysis (CFA)

CFA was conducted assuming the same factor structure as the original GAAIS. Unlike the original validation study (Schepman & Rodway, 2023), which employed the DWLS estimator, we used the robust maximum likelihood (MLR) estimator. Although our items were ordinal, we opted for MLR because our dataset was substantially larger and the response distribution approximated continuity. Prior simulation studies have shown that MLR performs comparably to DWLS for Likert-type scales with five or more response categories (Rhemtulla et al., 2012; Li, 2016), and it offers greater robustness to non-normality and missing data through full-information estimation. Initial model fit indices were: χ^2^ = 4371.523, df = 169, *p* < 0.001, CFI = 0.884, TLI = 0.87, NFI = 0.88, PNFI = 0.783, RFI = 0.865, IFI = 0.884, RNI = 0.884. However, RMSEA = 0.082 (90% CI = [0.08, 0.084]) and SRMR = 0.102 indicated suboptimal model fit. Item Neg3 (“Organizations use AI unethically”) showed low factor loading (0.17), suggesting poor alignment with the intended construct. Given its impact on convergent validity and model fit, Neg3 was removed, and the CFA was re-conducted (Table 1).

The revised model demonstrated improved fit: χ^2^ = 2163.885, df = 145, *p* < 0.001, CFI = 0.943, TLI = 0.932, NNFI = 0.932, NFI = 0.939, RFI = 0.928, IFI = 0.943, RNI = 0.943, RMSEA = 0.061 (90% CI = [0.059, 0.064]), and SRMR = 0.07. Reliability indicators were also strong (McDonald’s ω = 0.937; Cronbach’s α = 0.924). AVE for the first factor exceeded 0.50, while the second was acceptable (>0.40) (Table 2). Covariance was allowed between specific item pairs (e.g., Neg15 and Neg9; Pos7 and Pos18), which further improved model fit. The final model met all reliability and validity standards.

### 5.2. Construct Validity

Currently in Japan, no psychometric scale with established reliability and validity has been developed to measure general impressions of AI. However, with the recent advancement of AI technologies, scales that assess trust and perceptions toward generative AI have begun to receive attention. Katase (2021) developed the “AI Trust Scale” through a nationwide web survey and examined its reliability and validity. This scale consists of two subfactors: “distrust in AI’s fidelity” and “trust in AI’s social utility,” with reliability coefficients (α) of 0.73 and 0.72, respectively. The study concluded that further validation is necessary regarding the scale’s reliability and validity. In the present study, Katase’s (2021) instrument was used as one criterion to assess the external validity of the GAAIS-J. In addition, we examined whether the mean scores of each subscale (positive and negative attitudes) in the GAAIS-J correlated with scores on Katase’s two subfactors and the weekly frequency of generative AI use. Spearman’s rank correlation coefficient was used for the analysis (Table 3).

The GAAIS-J positive subscale showed a strong positive correlation with trust in AI (ρ = 0.617, *p* < 0.001) and a moderate correlation with generative AI usage frequency (ρ = 0.313, *p* < 0.001). The negative subscale showed a moderate negative correlation with AI distrust (ρ = −0.367, *p* < 0.001) and a weak but significant negative correlation with AI usage frequency (ρ = −0.093, *p* < 0.001). These findings support the scale’s external validity.

### 5.3. Socioeconomic Status (SES) and Gender Analysis

To examine the relationships between the GAAIS-J’s positive and negative factors and socioeconomic status (SES) as well as gender, correlation analyses were conducted using Spearman’s rank correlation coefficients (Table 4). SES indicators included age, income, and highest level of education. The analysis revealed no significant correlation between the positive factor and age (ρ = −0.012). However, significant positive correlations were observed with income (ρ = 0.188, *p* < 0.001) and education level (ρ = 0.051, *p* < 0.01), suggesting a modest association between more positive attitudes toward generative AI and higher income and educational attainment.

For the negative factor, a significant positive correlation was found with age (ρ = 0.033, *p* < 0.05), while significant negative correlations were observed with education (ρ = −0.284, *p* < 0.001) and income (ρ = −0.065, *p* < 0.001). These results suggest that negative attitudes toward generative AI may be more common among older individuals and those with lower levels of education, although the effect sizes were small.

Gender differences were observed only for the positive factor: men scored slightly higher than women, t(3687) = 5.663, *p* < 0.001, Cohen’s d = 0.189, 95% CI [0.124, 0.255] (small). No difference emerged for the negative factor, t(3687) = −0.521, *p* = 0.602, Cohen’s d = −0.017, 95% CI [−0.083, 0.048].

Taken together, these findings indicate that the positive factor of the GAAIS-J is primarily associated with income and education, with men exhibiting more favorable attitudes toward generative AI. In contrast, the negative factor was more strongly associated with older age and lower educational attainment, and showed no significant gender differences.

### 5.4. Big Five Personality Traits

To examine the associations between the positive and negative factors of the GAAIS-J and the Big Five personality traits (extraversion, agreeableness, conscientiousness, neuroticism, and openness), correlation analyses were conducted using Spearman’s rank correlation coefficients (Table 5).

The results showed that the positive factor of the GAAIS-J was significantly positively correlated with extraversion (ρ = 0.087, *p* < 0.001), agreeableness (ρ = 0.206, *p* < 0.001), conscientiousness (ρ = 0.302, *p* < 0.001), and openness (ρ = 0.302, *p* < 0.001). Among these, the strongest correlations were observed with openness and conscientiousness, suggesting that individuals with higher levels of these traits tend to have more positive attitudes toward generative AI. In contrast, the negative factor was significantly negatively correlated with agreeableness (ρ = −0.215, *p* < 0.001), conscientiousness (ρ = −0.231, *p* < 0.001), and openness (ρ = −0.191, *p* < 0.001), and showed no significant correlation with extraversion (ρ = −0.019, *p* = 0.243).

Notably, Neuroticism showed a distinct pattern: it was negatively correlated with the positive factor (ρ = −0.172, *p* < 0.001) and positively correlated with the negative factor (ρ = 0.141, *p* < 0.001). This suggests that individuals higher in Neuroticism tend to hold less positive and more negative attitudes toward generative AI, possibly reflecting heightened anxiety, distrust, or sensitivity to perceived risks associated with AI technologies. Unlike the other Big Five traits, which showed consistent directions of correlation across both factors, Neuroticism emerged as a potential dispositional risk factor for AI-related apprehension.

Taken together, these findings confirm that both the positive and negative factors of the GAAIS-J are meaningfully associated with the Big Five traits. These relationships suggest that individuals’ attitudes and trust toward AI may be influenced by their personality characteristics, with Neuroticism operating in a direction opposite to that of traits such as openness and conscientiousness.

## 6. Discussion

### 6.1. Cultural Adaptation and Item Evaluation

This study aimed to validate the reliability and construct validity of the Japanese version of the General Attitudes toward Artificial Intelligence Scale (GAAIS-J) through a large-scale web-based survey. The findings demonstrated significant associations between attitudes toward generative AI and individual differences, including socioeconomic status (SES), gender, and Big Five personality traits, supporting the predictive validity of the scale.

Item diagnostics revealed that item Neg3 (“Organizations use AI unethically”) showed weak loading on its intended factor. A plausible interpretation is that this item evokes culturally framed ethical concerns, which may diverge between Japanese and Anglophone contexts. Previous research suggests that Japanese individuals may emphasize relational or assurance-based trust over generalized trust (Yamagishi & Yamagishi, 1994), potentially reducing the salience of abstract institutional-ethics prompts.

The removal of Neg3 was thus based not only on its weak psychometric performance but also on its potential for cultural non-equivalence. In Japan, public trust in institutions tends to be higher and perceptions of AI more technophilic (Nomura et al., 2006), contrasting with more skeptical views prevalent in Western contexts (Pew Research Center, 2025). Recent global surveys suggest that responses to questions about ethical AI misuse often reflect institutional trust rather than AI-specific concerns (Gillespie et al., 2025; Rieger et al., 2021). Consequently, Neg3 may conflate cultural differences in institutional cynicism and attitudes toward AI itself. Its exclusion enhances construct validity and aligns with cross-cultural adaptation guidelines that recommend removing items with interpretive inequivalence (F. Van de Vijver & Tanzer, 2004).

Furthermore, a comparison with the original English GAAIS (Schepman & Rodway, 2023) indicates that the Japanese adaptation maintained the same two-factor structure and comparable reliability indices. Apart from the exclusion of one culturally inconsistent item (Neg3), the pattern of factor loadings and the distinction between positive and negative attitudes were consistent with those reported in the original validation. These results suggest that the GAAIS-J preserves the theoretical and psychometric integrity of the source instrument while achieving cultural and linguistic equivalence.

### 6.2. Model Evaluation and Construct Validity

In line with structural equation modeling guidelines (Byrne, 2016; Kline, 2023), modification indices (MIs) were examined. A small number of theoretically justified residual covariances were introduced between semantically similar items (Brown, 2015). These additions, which do not alter the latent factor structure, addressed method variance while preserving theoretical coherence. All covariances were guided by conceptual rationale, adhering to psychometric standards for model parsimony and theoretical justification (MacCallum et al., 1992; Hair et al., 2019).

The scale’s external validity was supported through correlations with variables such as SES, personality traits, and general AI usage, which align with well-established frameworks. For instance, the Technology Acceptance Model (TAM) and UTAUT frameworks predict that positive AI attitudes relate to greater perceived usefulness and actual use (Davis, 1989). Observed associations between personality traits (e.g., openness, conscientiousness) and AI attitudes reflect broader findings linking personality to digital adoption in organizational contexts. Trust in AI, as a multidimensional construct encompassing both affective and cognitive components (Glikson & Woolley, 2020), is echoed in the scale’s bifactorial structure.

### 6.3. Generalizability, Limitations, and Future Directions

Socio-demographic gradients such as age and education mirrored typical digital-divide patterns, yet these results should be interpreted with caution. Given the large sample size, even very small effects reached statistical significance. To address this, interpretations were calibrated according to effect sizes, and we recommend that future studies supplement *p*-values with confidence intervals (Putnick & Bornstein, 2016).

Although alternative structural specifications (e.g., higher-order or bifactor models) could be examined, they were intentionally excluded in order to maintain structural fidelity with the original GAAIS (Schepman & Rodway, 2023). As emphasized by Borsa et al. (2012), cross-cultural validation should prioritize construct equivalence, and introducing new model forms can risk conceptual drift.

The AVE for the negative factor fell slightly below the conventional 0.50 threshold (Fornell & Larcker, 1981), a result that is common for scales encompassing items with diverse content (Hair et al., 2019). This factor includes elements ranging from technical malfunction to distrust, which may naturally reduce inter-item correlations. Nevertheless, all factor loadings were significant and theoretically coherent. Discriminant validity was supported by distinct correlation patterns between the two factors and external constructs (e.g., general trust, AI-specific distrust, and Big Five traits). Although future research might further explore bifactor or method-factor models, the present approach ensures cross-cultural comparability and remains faithful to the original validation framework.

While a multi-group CFA was not conducted due to scope constraints, future studies should test measurement invariance across demographic subgroups. Likewise, behavioral validation (e.g., usage logs) could provide deeper insight into the dynamics between attitudes and actual AI use.

Finally, although the large-scale online sample afforded high statistical power, its nonprobabilistic nature limits generalizability. Future research should employ probability sampling, preregistered invariance testing, and mixed-methods approaches to enhance robustness. Incorporating behavioral data would also help elucidate the attitude–behavior relationships central to technology acceptance models (Davis, 1989).

## 7. Conclusions

The GAAIS-J reproduced the original two-factor structure with satisfactory fit and reliability, supporting its use for assessing public attitudes toward AI in Japan. Situating the findings within established frameworks, our results are consistent with multidimensional views of AI trust and with technology-acceptance theories linking favorable attitudes to self-reported use (Davis, 1989; Glikson & Woolley, 2020; Venkatesh et al., 2003). Methodologically, we prioritized structural fidelity to the source instrument over exploratory re-specification, in line with cross-cultural adaptation guidance. As a planned extension, we will examine multi-group measurement invariance (configural → metric → scalar) across gender and age groups—and, ideally, across languages—to confirm that observed differences reflect substantive attitudes rather than measurement artifacts (Borsa et al., 2012; Putnick & Bornstein, 2016).

Tools such as the GAAIS-J can inform evaluation of educational and workplace interventions, public communication, and ethical governance of AI. To enhance applied value, future studies should report effect sizes and confidence intervals, integrate objective usage indicators, and test invariance to support fair cross-group/cross-national comparisons.

## Figures and Tables

**Table 1:** Factor loadings for CFA1.

	Item	Standardized Estimate	SE	z	*p*	95% CI Lower	95% CI Upper
Positive							
Pos1	For routine transactions, I would rather interact with an artificially intelligent system than with a human.	0.645	0.018	35.942	<0.001	0.609	0.680
Pos2	Artificial Intelligence can provide new economic opportunities for this country.	0.712	0.016	45.736	<0.001	0.682	0.743
Pos4	Artificially intelligent systems can help people feel happier.	0.694	0.014	48.231	<0.001	0.666	0.722
Pos5	I am impressed by what Artificial Intelligence can do.	0.775	0.014	53.701	<0.001	0.747	0.804
Pos7	I am interested in using artificially intelligent systems in my daily life.	0.815	0.014	58.027	<0.001	0.787	0.842
Pos11	Artificial Intelligence can have positive impacts on people’s wellbeing.	0.686	0.014	47.647	<0.001	0.658	0.715
Pos12	Artificial Intelligence is exciting.	0.672	0.015	43.574	<0.001	0.642	0.702
Pos13	An artificially intelligent agent would be better than an employee in many routine jobs.	0.567	0.018	30.685	<0.001	0.531	0.604
Pos14	There are many beneficial applications of Artificial Intelligence.	0.600	0.016	36.812	<0.001	0.568	0.632
Pos16	Artificially intelligent systems can perform better than humans.	0.564	0.017	32.728	<0.001	0.530	0.598
Pos17	Much of society will benefit from a future full of Artificial Intelligence.	0.666	0.015	45.609	<0.001	0.637	0.694
Pos18	I would like to use Artificial Intelligence in my own job.	0.748	0.015	48.910	<0.001	0.718	0.778
Negative							
Neg6	I think artificially intelligent systems make many errors.	0.394	0.020	19.545	<0.001	0.355	0.434
Neg8	I find Artificial Intelligence sinister.	0.659	0.016	40.515	<0.001	0.627	0.691
Neg9	Artificial Intelligence might take control of people.	0.754	0.018	40.797	<0.001	0.718	0.791
Neg10	I think Artificial Intelligence is dangerous.	0.795	0.016	51.254	<0.001	0.765	0.826
Neg15	I shiver with discomfort when I think about future uses of Artificial Intelligence.	0.714	0.018	39.064	<0.001	0.679	0.750
Neg19	People like me will suffer if Artificial Intelligence is used more and more.	0.637	0.019	33.401	<0.001	0.600	0.675
Neg20	Artificial Intelligence is used to spy on people.	0.522	0.019	26.939	<0.001	0.484	0.560
Neg3	Organizations use Artificial Intelligence unethically.	0.170	0.023	7.439	<0.001	0.125	0.215

**Table 2:** Factor loadings for CFA2.

	Item	Standardized Estimate	SE	z	*p*	95% CI Lower	95% CI Upper
Positive							
Pos1	For routine transactions, I would rather interact with an artificially intelligent system than with a human.	0.587	0.014	41.584	<0.001	0.559	0.614
Pos2	Artificial Intelligence can provide new economic opportunities for this country.	0.750	0.010	72.191	<0.001	0.729	0.770
Pos4	Artificially intelligent systems can help people feel happier.	0.773	0.009	82.918	<0.001	0.755	0.791
Pos5	I am impressed by what Artificial Intelligence can do.	0.774	0.009	88.499	<0.001	0.757	0.791
Pos7	I am interested in using artificially intelligent systems in my daily life.	0.771	0.009	88.279	<0.001	0.754	0.788
Pos11	Artificial Intelligence can have positive impacts on people’s wellbeing.	0.793	0.010	83.351	<0.001	0.774	0.812
Pos12	Artificial Intelligence is exciting.	0.723	0.011	63.264	<0.001	0.700	0.745
Pos13	An artificially intelligent agent would be better than an employee in many routine jobs.	0.590	0.016	36.845	<0.001	0.558	0.621
Pos14	There are many beneficial applications of Artificial Intelligence.	0.706	0.013	53.264	<0.001	0.680	0.732
Pos16	Artificially intelligent systems can perform better than humans.	0.618	0.015	39.933	<0.001	0.588	0.649
Pos17	Much of society will benefit from a future full of Artificial Intelligence.	0.769	0.011	70.059	<0.001	0.747	0.790
Pos18	I would like to use Artificial Intelligence in my own job.	0.678	0.011	60.372	<0.001	0.656	0.699
Negative							
Neg6	I think artificially intelligent systems make many errors.	0.431	0.021	20.208	<0.001	0.389	0.473
Neg8	I find Artificial Intelligence sinister.	0.752	0.014	52.104	<0.001	0.723	0.780
Neg9	Artificial Intelligence might take control of people.	0.679	0.017	39.502	<0.001	0.646	0.713
Neg10	I think Artificial Intelligence is dangerous.	0.791	0.012	66.274	<0.001	0.768	0.815
Neg15	I shiver with discomfort when I think about future uses of Artificial Intelligence.	0.699	0.016	43.997	<0.001	0.668	0.730
Neg19	People like me will suffer if Artificial Intelligence is used more and more.	0.624	0.017	36.833	<0.001	0.591	0.658
Neg20	Artificial Intelligence is used to spy on people.	0.534	0.018	29.065	<0.001	0.498	0.570

Note. CFI = Comparative Fit Index; TLI = Tucker–Lewis Index (also known as NNFI = Non-Normed Fit Index); NFI = Normed Fit Index; RFI = Relative Fit Index; IFI = Incremental Fit Index; RNI = Relative Noncentrality Index; RMSEA = Root Mean Square Error of Approximation; SRMR = Standardized Root Mean Square Residual; AVE = Average Variance Extracted.

**Table 3:** Correlations between the GAAIS and the subscales of the AI Trust Scale.

	How Often Do You Use Generative AI in a Typical Week?	Trust in AI’s Social Utility	Distrust in AI’s Fidelity
GAAIS Pos	0.313 ***	0.617 ***	−0.094 ***
GAAIS Neg	−0.093 ***	−0.367 ***	0.400 ***

*** *p* < 0.001.

**Table 4:** Correlations between the GAAIS and socioeconomic status (SES).

	Age	Income	Educational Level
GAAIS Pos	−0.012	0.188 ***	0.161 ***
GAAIS Neg	0.033 *	−0.065 ***	0.067 ***

* *p* < 0.05, *** *p* < 0.001.

**Table 5:** Correlations between the GAAIS and the Big Five personality traits.

	Openness	Conscientiousness	Extraversion	Agreeableness	Neuroticism
GAAIS Pos	0.158 ***	0.166 ***	0.087 ***	0.206 ***	−0.172 ***
GAAIS Neg	−0.039 *	−0.097 ***	−0.019	−0.215 ***	0.141 ***

* *p* < 0.05, *** *p* < 0.001.

## Data Availability

Anonymized data and analysis code that support the findings of this study are available from the corresponding author upon reasonable request.
